# Identification and Analysis of the Putative Pentose Sugar Efflux Transporters in *Escherichia coli*

**DOI:** 10.1371/journal.pone.0043700

**Published:** 2012-08-28

**Authors:** Khushnuma Koita, Christopher V. Rao

**Affiliations:** Department of Chemical and Biomolecular Engineering, University of Illinois at Urbana-Champaign, Urbana, Illinois, United States of America; University of Groningen, The Netherlands

## Abstract

*Escherichia coli* possesses a number of proteins that transport sugars out of the cell. We identified 31 candidate sugar efflux transporters based on their similarity to known sugar efflux transporters. We then tested whether these transporters affect arabinose and xylose metabolism. We identified 13 transporters – *setC, cmr, ynfM, mdtD, yfcJ, yhhS, emrD*, *ydhC, ydeA*, *ybdA*, *ydeE*, *mhpT*, and *kgtP –* that appeared to increase or decrease intracellular arabinose concentrations when respectively deleted or over-expressed. None of the candidate transporters affected xylose concentrations. These results indicate that *E. coli* possesses multiple arabinose efflux transporters. They also provide a novel target for future metabolic engineering.

## Introduction

*Escherichia coli* expresses proteins that not only transport sugars into cells but also a number of proteins that transport them out of the cell. The canonical example is the SET family of transporters: SetA, SetB, and SetC [1], [2]. These transporters have been previously shown to actively efflux glucose and lactose from cells. In addition, they have broad specificity for various glucosides or galactosides. Aside from the SET family, YdeA has also been shown to efflux L-arabinose from cells [3], [4].

Efflux transporters have been extensively studied in the literature in the context of multi-drug resistance (MDR). Little is known about these sugar efflux transporters, or what roles they may play in cellular metabolism. One possible mechanism is to relieve sugar-phosphate stress. Sun and Vanderpool demonstrated that SetA participates in the glucose-phosphate stress response [5]. However, they found that SetA does not efflux alpha-methylglucoside, the nonmetabolizable sugar analog used to elicit the response. Another proposal is that they function as safety valves, preventing excess sugar from accumulating within the cell [6]. However, this hypothesis has yet to be tested.

In this work, we investigated L-arabinose and D-xylose (hereafter referred to simply as arabinose and xylose, respectively) efflux in *E. coli*. Sequence analysis identified 31 candidate sugar efflux transporters in *E. coli* based on homology to known sugar efflux transporters. Using genetic approaches, we tested whether these candidate efflux transporters target arabinose and xylose. We were able to identify multiple putative arabinose efflux transporters but interestingly none for xylose.

## Results

### Effect of Deleting Candidate Sugar Efflux Transporters on Arabinose Metabolism

We identified 31 candidate sugar efflux transporters in *E. coli* based on homology to the known sugar efflux transporters: *ydeA*, *setA*, and *setB* (**Table 1**). We identified these homologs based on their membership in the same Cluster of Orthologous Group of proteins, COG2814 [7], [8]. These homologs included a number of multidrug efflux transporters, both known and putative, along with five transporters involved in the uptake of various metabolites (*shiA*, *kgtP*, *galP*, *nanT*, and *proP*).

**Table 1:** List of genes.

Gene	Annotation
setA	broad specificity sugar efflux system
*setB*	lactose/glucose efflux system
*setC*	predicted sugar efflux system
*ydeA*	sugar efflux transporter
*mhpT*	putative 3-hydroxyphenylpropionic acid transporter
*yajR*	putative transporter
*ybdA (entS)*	predicted transporter
*cmr (mdfA)*	multidrug efflux system protein
*ycaD*	putative MFS family transporter
*yceL*	orf, hypothetical protein
*ydeE*	predicted transporter
*ynfM*	predicted transporter
*ydhP*	predicted transporter
*mdtG (yceE)*	predicted drug efflux system
*yebQ*	putative transporter
*shiA*	shikimate transporter
*mdtD (yegB)*	multidrug efflux system protein
*bcr*	bicyclomycin/multidrug efflux system
*yfcJ*	predicted transporter
*kgtP*	alpha-ketoglutarate transporter
*ygcS*	putative transporter
*galP*	D-galactose transporter
*nanT*	sialic acid transporter
*yhhS*	putative transporter
*nepI (yicM)*	predicted transporter
*emrD*	2-module integral membrane pump; multidrug resistance
*mdtL (yidY)*	multidrug efflux system protein
*proP*	proline/glycine betaine transporter
*yjhB*	putative transporter
*yjiO (mdtM)*	multidrug efflux system protein
*ydhC*	predicted transporter

If any of these genes encodes an efflux transporter of arabinose, then deleting the gene should increase the intracellular sugar concentration of arabinose. As an indirect measure of intracellular arabinose concentrations, we employed transcriptional fusions of *araBAD* promoter to Venus, a fast-folding variant of the yellow fluorescent protein [9]. The average activity of the *araBAD* promoter as determined using fluorescence is proportional to extracellular arabinose concentrations and presumably relative intracellular arabinose concentrations as well based on the known mechanism for regulation [10]. To test the effect of deleting these genes, we grew the cells in tryptone broth at varying concentrations of arabinose and then harvested the cells at late-exponential phase to determine relative *araBAD* promoter activities. The resulting data were fit to a Michaelis-Menten dose-response curve with the governing parameters, V_max_ and K_m_, reported (**Table 2**).

**Figure 1:**
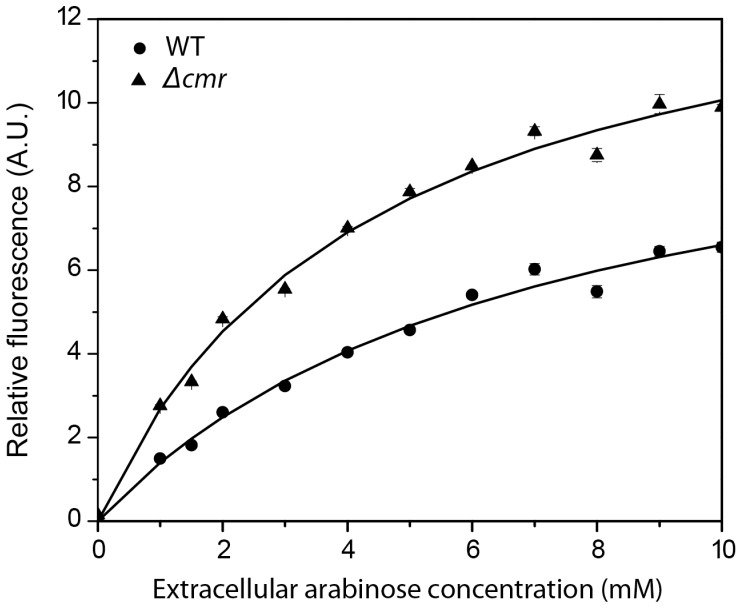
Dose-response curve for the *Δcmr* mutant. P*_araB_*-*venus* in WT and *Δcmr* mutant strains were measured for fluorescence and the results are reported as Fluorescence/OD_600_. Strains were induced with arabinose concentrations varying from 0 mM to 10 mM.

Deletion of the following eight genes was found to increase intracellular arabinose concentrations as compared to the wild-type control: *setC, cmr, ynfM, mdtD, yfcJ, yhhS, emrD* and *ydhC*. Deleting the other genes had no effect, with the dose-response curves indistinguishable from the wild-type control. Our specific metric was a statistically significant difference in the calculated V_max_ values. A representative dose-response curve for the *Δcmr* mutant is shown in **Figure 1**. These results suggest that these eight genes encode arabinose efflux transporters. Among them, only SetC has previously been implicated in sugar efflux based on its homology to SetA and SetB, two transporters known to efflux lactose [2]. Cmr, also known as MdfA, and EmrD are known multidrug efflux transporters [11], [12], [13]. Over-expression of Cmr has also been shown to limit the uptake of isopropyl-β-D-thiogalactopyranoside (IPTG), an effect attributed to efflux [14]. YnfM, MdtD, YfcJ, and YdhC are uncharacterized major facilitator superfamily (MFS) transporters. YhhS is also an uncharacterized MFS transporter that has been implicated glyphosate resistance [15].

**Table 2:** K_M_ and V_max_ values* determined from deletion and overexpression studies.

Gene	K_M_	V_max_		Gene	2 mMIPTG		0.2 mMIPTG	
					**K_M_**	**V_max_**	**K_M_**	**V_max_**
Control	3.17 ± 1.25	6.88 ± 1.01		Control	2.73 ± 1.01	11.1 ± 1.01	2.84 ± 1.30	11.9 ± 1.92
*setC*	3.51 ± 0.96	12.7 ± 1.37		*setC*	Growth defect		5.27 ± 3.86	6.49 ± 2.22
*cmr*	3.94 ± 1.36	13.2 ± 1.88		*ydeE*	Growth defect		4.10 ± 0.93	7.59 ± 0.72
*ynfM*	3.77 ± 0.96	12.3 ± 1.28		*ydeA*	3.11 ± 1.09	4.53 ± 0.59	No effect	
*mdtD*	4.17 ± 1.56	13.8 ± 2.18		*kgtP*	2.75 ± 2.67	7.65 ± 2.60	No effect	
*yfcJ*	3.04 ± 1.95	9.96 ± 2.34		*mhpT*	0.34 ± 0.17	7.08 ± 0.36	1.35 ± 0.71	7.47 ± 0.97
*yhhS*	4.96 ± 1.97	14.8 ± 2.67		*ybdA*	0.93 ± 0.35	6.84 ± 0.52	1.33 ± 0.75	7.93 ± 1.08
*emrD*	3.61 ± 1.48	12.6 ± 2.05						
*ydhC*	3.82 ± 1.61	12.4 ± 2.12						

* K_M_ values are reported in mM. V_max_ values are reported as Fluorescence/OD_600_. Errorbars provide 95% confidence intervals on the parameter estimates.

Interestingly, we did not observe any change in a Δ*ydeA* mutant, though we did observe an effect when overexpressing *ydeA* as discussed below. This gene is known to encode an arabinose efflux transporter [3], [4], and Carolé and coworkers have previously shown that disrupting the *ydeA* gene increases intracellular arabinose concentrations. The lack of agreement between their results and ours may be due to the fact that they employed a strain lacking the arabinose metabolic genes, which dramatically increases intracellular arabinose concentrations as shown below, whereas we employed wild-type MG1655.

### Effect of Over-expressing Candidate Sugar Efflux Transporters on Arabinose Metabolism

We also over-expressed these candidate sugar efflux transporters. If these genes encode arabinose efflux transporters, then over-expressing them should reduce intracellular arabinose concentrations. To express these genes, they were cloned on high-copy plasmids under the control of the strong, IPTG-inducible *trc* promoter. Once again, transcriptional fusions to the *araBAD* promoter were employed as indirect measures of intracellular arabinose and the results were recorded in terms of the governing parameters for a Michaelis-Menten dose-response curve (**Table 2**).

**Figure 2:**
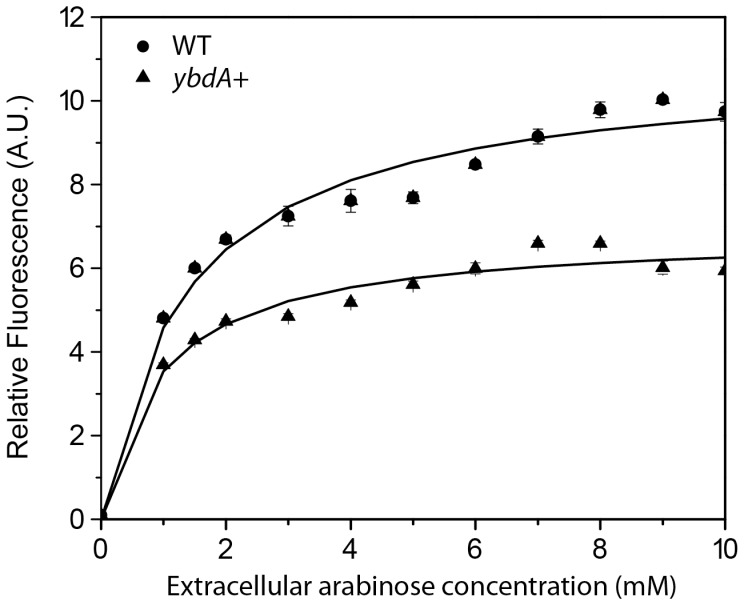
Dose-response curve for *ybdA* overexpression. P*_araB_*-*venus* in WT and *ybdA* overexpressing strains were measured for fluorescence and the results are reported as Fluorescence/OD_600_. Strains were induced with arabinose concentrations varying from 0 mM to 10 mM. The *ybdA* overexpressing strain was induced with 2 mM IPTG.

Over-expression of six genes were found to reduce intracellular arabinose concentrations: *setC*, *ydeA*, *ybdA*, *ydeE*, *mhpT*, and *kgtP*. A representative dose-response curve for *ybdA* is given in **Figure 2**. Among the six, only *setC* and *ydeA* have been implicated in sugar efflux as discussed above. YbdA, also known as EntS, has been implicated in enterobacterin transport [16] and is involved in the resistance to multiple chemical stresses [17]. YdeE is an uncharacterized MFS transporter involved in dipeptide transport and resistance [18]. MhpT is a putative 3-hydroxyphenylpropionic acid transporter. KgtP, interestingly, is known to be α ketoglutarate transporter [19].

When comparing these results with our deletion results, only *setC* gave a consistent result where loss of the gene increases intracellular arabinose and overexpression decreases it. Over-expression of *yfcJ*, *yhhS*, *emrD*, and *ydhC* had no effect on intracellular arabinose concentrations. Three genes that gave a deletion phenotype were toxic to the cell when over-expressed: *cmr*, *ynfM*, and *mdtD*.

Overall, 12 genes were toxic to *E. coli* when over-expressed: *setB*, *setC, yajR, cmr, ycaD, ydeE, ynfM, mdtD, bcr, ygcS, yjhB,* and *yjiO*. To limit toxicity, the over-expression experiments were performed at two concentrations of the IPTG inducer. However, six genes were still toxic when induced even at lower concentrations of IPTG: *ycaD, ynfM, mdtD, bcr, and ygcS,* and *yjhB*. Both *setC* and *ydeE* were toxic to the cell when expressed using the higher IPTG concentration; only at the lower concentration did we observe an effect. Conversely, we only observed an effect with *ydeA* and *kgtP* when they were induced at higher IPTG concentration. Both *ybdA* and *mphT* decrease intracellular arabinose concentrations at both induction levels.

### Xylose is Not a Target for Any of the Candidate Efflux Transporters

We also tested whether any of these candidate efflux transporters target xylose. A similar approach was employed as before except that we used transcriptional fusions of the *xylA* promoter to Venus. The *xylA* promoter is positively regulated by XylR in response to xylose [20] and can equivalently be used to determine relative intracellular xylose concentration. Otherwise, the experiments were performed in an identical manner to the arabinose experiments. Despite the similarities between xylose and arabinose, we did not observe any change in intracellular xylose concentrations as compared to the wild-type control when any of candidate genes were deleted or overexpressed.

### Arabinose is a Substrate for Most Efflux Transporters

The arabinose metabolic intermediate, L-ribulose-5-phosphate, is toxic to *E. coli* [21]. This may provide one possible explanation as to why arabinose may be effluxed from cells. It also suggests that arabinose itself may not be the target for efflux but rather an intermediate of arabinose metabolism. To test this hypothesis, we blocked different steps in arabinose metabolism by deleting the cognate gene and then determined how the mutation would affect our results concerning arabinose efflux. If arabinose is not the target for these transporters and the downstream metabolite is instead, then we expect that blocking metabolism will reduce the effect of these transporters. Briefly, the arabinose metabolic pathway involves three steps: 1) isomerization to L-ribulose (*araA*); 2) phosphorylation to L-ribulose-5-phosphate (*araB*); and 3) inversion to D-xylulose-5-phosphate (*araD*). We tested three mutants: Δ*araA*, Δ*araB*, and Δ*araBAD*. We did not test a Δ*araD* mutant as arabinose is toxic in this strain.

Blocking any step in arabinose metabolism significantly increased the concentration of intracellular arabinose relative to the wild-type control. This is expected, as arabinose is no longer being metabolized in any of these strains. However, we did not observe any further increase when *setC, mhpT, cmr, ynfM, mdtD, yfcJ, yhhS,* and *emrD* were deleted as well (data not shown). These results suggest that arabinose concentrations are already high in these cells and eliminating efflux does not further increase arabinose concentration. Therefore, we were unable to conclude anything from these deletion experiments.

**Figure 3:**
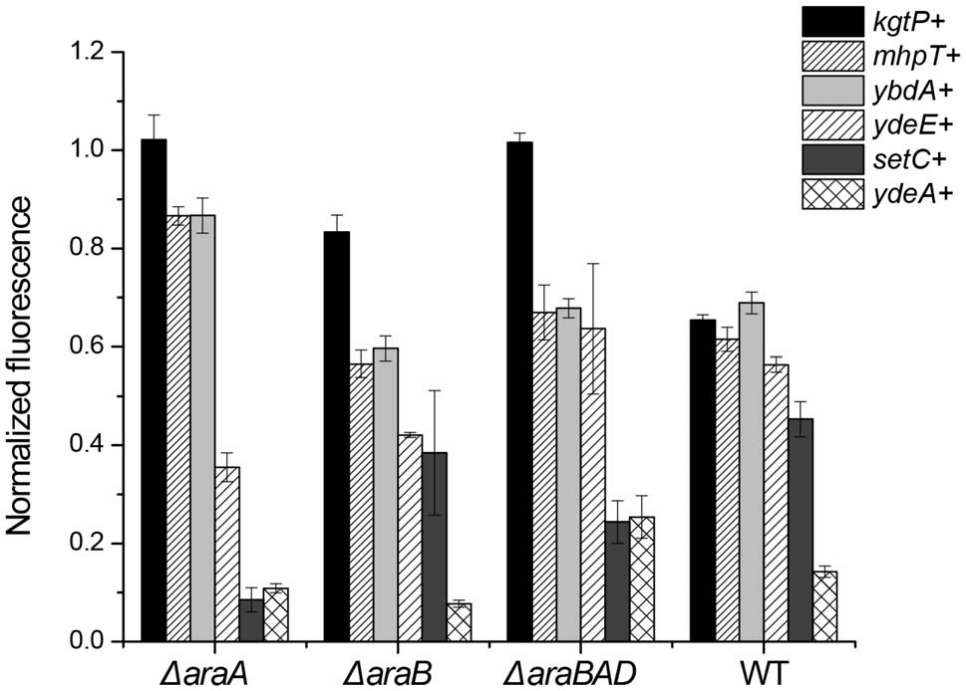
Overexpression of the efflux transporters in arabinose metabolic mutants. The data is normalized to the relative fluorescence values of the control strains (P*_araB_*-*venus* and pTrC99A in each of the individual arabinose mutants). All of the strains were induced with 5 mM arabinose. Overexpression of the the efflux transporters was induced with 2 mM IPTG except for the overexpression of *setC* and *ydeE* where 0.2 mM IPTG was used.

The effect of overexpressing of *setC, ydeE,* and *ydeA* was most pronounced in a Δ*araA* mutant, indicating that the three target arabinose (**Figure 3**). If they targeted a downstream metabolite, then we would not expect a reduction in intracellular arabinose concentrations. The data for *mhpT* and *ybdA* were more equivocal though again they suggest that these transporters both target arabinose. Interestingly, we only observed a reduction in relative arabinose concentrations only when *kgtP* was overexpressed in a Δ*araB* mutant or the wild type. These results suggest that L-ribulose may be the target for KgtP as we did not observe a reduction relative to the wild-type control in a Δ*araA* mutant where the downstream metabolites are not produced. Note, the results in Figure 3 are normalized relative to the wild-type control.

### Expression of Efflux Transporters is Induced by Arabinose

The last question explored was whether arabinose induced the expression of these transporters. To answer this question, we fused the promoters for these transporters to Venus and then tested whether they were activated by arabinose. Of the 13 transporters, we were able to construct functional promoter fusions to all but five. Our transcriptional fusions to the *mhpT*, *mdtD,* and *yfcJ* promoters were not functional despite repeated attempts. In addition, the fusions to the *setC* and *ydhC* promoters were very weak and not included in our results as a consequence.

**Figure 4:**
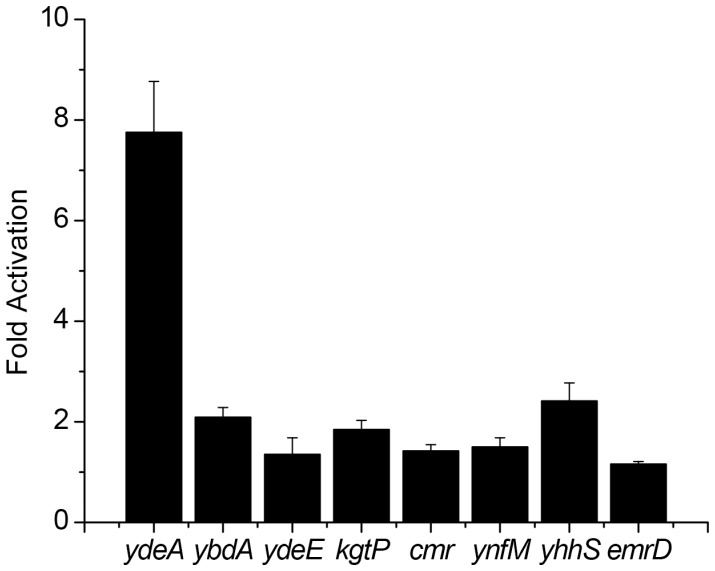
Efflux promoter transcriptional fusions. Reporters are in a wild-type background and the data are normalized to the relative fluorescence values when no arabinose is added. All of the strains were induced with 10 mM arabinose.

Of the eight remaining functional promoters, only the *ydeA* and *yhhS* promoters were activated at significant levels (**Figure 4**). The remaining six promoters were also activated by arabinose though the effect is less than two fold. How these promoters, in particular for the *ydeA* and *yhhS* genes, are being activated by arabinose is not known. Interesting, *ydeA* is strongly induced by arabinose yet deleting this gene does not affect intracellular arabinose concentrations – only when over-expressed do we observe an effect.

## Discussion

We identified multiple candidate sugar efflux transporters in *E. coli* based on sequence homology to known sugar efflux transporters and then tested to see whether they target arabinose and xylose. Thirteen putative arabinose efflux transporters were identified based on their ability to alter intracellular arabinose concentration. Eight were found to increase intracellular arabinose concentrations when deleted and six to decrease concentrations when overexpressed. Only one transporter, SetC, was found to yield reciprocal results when deleted or overexpressed. Among the 13 putative transporters, only YdeA has previously been shown to be an arabinose efflux transporter. Interestingly, none affected intracellular xylose concentrations.

We cannot definitely say whether these putative transporters in fact efflux arabinose as we did not directly measure arabinose transport. All that we can say with certainty is that these 13 transporters inhibit the accumulation of arabinose with efflux being the likely but not sole possible mechanism. Alternate mechanisms include the inhibition of the arabinose uptake and the efflux of other compounds that affect arabinose metabolism or possibly stimulate other efflux transporters.

Many efflux transporters are TolC dependent [22]. Martin and Rosner previously found that the *mar*/*sox*/*rob* regulon was activated in a *tolC* mutant [23]. They proposed that this activation is due to the accumulation of intracellular metabolites due to the loss of efflux. We also tested whether any of the identified arabinose efflux transporters were TolC dependent. While we observed that intracellular arabinose concentrations were higher in a *tolC* mutant, we were unable to determine whether any of these efflux transporters were TolC dependent as our data were equivocal (results not shown). Aside from TolC, these candidate efflux transporters may associate with other proteins that were not considered in our analysis. If this is the case, then the over-expression studies may have missed some efflux transporters as the accessory proteins would not be expressed in the correct stoichiometry for these over-expressed transporters to be functional.

A number of the identified efflux transporters have previously been shown to transport other compounds both in and out of the cell. Cmr, for example, is known to efflux a number of chemically unrelated compounds [11], so it is not entirely implausible that it effluxes arabinose as well. This is consistent with multidrug efflux transporters in general, which are known to have broad substrate specificities. What is surprising is that transporters such as KgtP, a known α ketoglutarate transporter [19], also inhibit arabinose uptake and perhaps efflux it as well. Clearly further research is required to identify the exact mechanisms for how these transporters affect arabinose uptake and efflux.

Why would *E. coli* want to efflux arabinose from the cell? One possibility is to limit the accumulation of toxic sugar phosphates, such as the arabinose metabolic intermediate L-ribulose-5-phosphate, within the cell. If the flux of arabinose is greater than the pentose phosphate pathway can accommodate, then L-ribulose-5-phosphate may accumulate within the cell due to an effective roadblock within the pathway. Under such a scenario, arabinose efflux transporters would provide a relief valve for the cell. A second possibility is to limit the accumulation of methylglyoxal, another toxic compound to *E. coli* [24]. Methylglyoxal synthase is thought to provide a relief valve in glycolysis, preventing the buildup of dihydroxyacetone phosphate when inorganic phosphate is limiting. If the flux of arabinose is too high, it may overwhelm the glycolytic branch of the pathway leading to a buildup of dihydroxyacetone phosphate. Consistent with this model, the addition of cAMP to cells grown on arabinose or xylose, which increases the uptake of these two sugars, produce excess methylglyoxal, arresting cell growth [25]. Interestingly, the effect is less severe with arabinose, consistent with our result showing a lack of xylose efflux transporters. In addition, the overexpression of methylglyoxal synthase is lethal to cells grown on arabinose and xylose but not on glucose. Unlike arabinose and xylose dissimilation, glycolysis is linked to glucose transport through the phosphenolpyruvate transferase system [26]. In the case of arabinose and xylose dissimilation, transport and metabolism are uncoupled. Arabinose efflux may provide a relief valve to accommodate this uncoupling. Why there are not similar relief valves for xylose is not known, though it may be that xylose efflux transporters do in fact exist but were not among the 31 analyzed.

Other outstanding questions concern the regulation of these transporters. Arabinose was found to induce the expression of many efflux transporters, where the effects were most pronounced for the YdeA and YhhS transporters. We and others have previously speculated that the multidrug antibiotic resistance (MDR) mechanisms in bacteria evolved in part as a mechanism to prevent the buildup of toxic metabolites [23], [27]. In fact, we found that 2,3-dihydroxybenzoate, an intermediate in enterobactin biosynthesis, directly binds to and inactivates MarR, the key regulator of the *marRAB* operon involved in antibiotic resistance. Whether arabinose and other sugars activate the MDR regulators is not known though it is an intriguing hypothesis nonetheless.

We conclude by noting that sugar efflux may provide a novel target for metabolic engineering. In particular, one may be able to increase the metabolic flux through a given pathway by eliminating the efflux of the governing sugar. The present work was motivated in part by looking at novel targets for optimizing pentose metabolism in *E. coli*. Of course, naively removing these transporters may have a detrimental effect on the cell due to the buildup of toxic metabolites. Any design targeting efflux may also need to make commensurate changes to transport and metabolism. Whether sugar efflux transporters provide a viable target for metabolic engineering is not known, but it does provide a totally unexplored area that clearly merits further investigation.

## Materials and Methods

### Media and Growth Conditions

Luria-Bertani liquid and solid medium (10 g/l tryptone, 5 g/l yeast extract, 10 g/l NaCl) was used for routine bacterial culture and genetic manipulation. All experiments were performed in tryptone broth at 37**°**C. Antibiotics were used at the following concentrations: ampicillin at 100 µg/ml, chloramphenicol at 20 µg/ml, and kanamycin at 40 µg/ml. Inducer isopropyl-β-D-galactopyranoside (IPTG) was used at a concentration of 2 mM unless otherwise specified. All experiments involving the growth of cells carrying the helper plasmid pKD46 were performed at 30°C. Loss of pKD46 was achieved by growth at 42°C under nonselective conditions on LB agar. The removal of the antibiotic cassette from the FLP recombinant target (FRT)-chloramphenicol/kanamycin-FRT insert was obtained by the transformation of the helper plasmid pCP20 into the respective strain and selection on ampicillin at 30°C. Loss of pCP20 was obtained by growth at 42°C under nonselective conditions on LB agar. Primers were purchased from IDT, Inc. Enzymes were purchased from New England Biolabs and Fermentas.

### Strain and Plasmid Construction

Bacterial strains and plasmids used in this study are described in **Tables 3** **and** **4**. All strains are isogenic derivatives of *Escherichia coli* K-12 strain MG1655. All cloning steps were performed in *E. coli* strain DH5α (*phi-80d lac*Δm15 *enda1 recA1 hsdR17 supE44 thh*-1 *gyrA96 relA*Δ*lacU169*). Targeted gene deletions and subsequent marker removal were made using the λRed recombinase method of Datsenko and Wanner [28]. The generalized transducing phage P1*vir* was used in all genetic crosses according to standard methods [29].

**Table 3:** Bacterial strains used in this work.

Strain	Genotype or relevant characteristics^a^	Source or referenceb,c
MG1655	F^-^ λ^-^ *ilvG rph-1*	CGSC #7740
DH5α	*phi-80d lac*Δm15 *enda1 recA1 hsdR17 supE44 thh*-1 *gyrA96 relA*Δ*lacU169*	New England Biolabs
CR400	Δ*araA*::*kan*	[32]
CR401	Δ*araB*::*kan*	[32]
CR404	Δ*araBAD*::*kan*	[32]
CR701	Δ*tolC*::*cat*	[27]
CR702	Δ*tolC*::FRT	[27]
CR1100	Δ*setA*:: FRT *kan* FRT (77621–78799)	
CR1101	Δ*setB*:: FRT *kan* FRT (2261885–2263066)	
CR1102	Δ*setC*:: FRT *kan* FRT (3834976–3836160)	
CR1103	Δ*ydeA*:: FRT *kan* FRT (1615052–1616242)	
CR1104	Δ*mhpT*:: FRT *kan* FRT (374683–375894)	
CR1105	Δ*yajR*:: FRT *kan* FRT (444526–445890)	
CR1106	Δ*ybdA*::FRT *kan* FRT (621523–622773)	
CR1107	Δ*cmr*::FRT *kan* FRT (882896–884128)	
CR1108	Δ*ycaD*::FRT *kan* FRT (945094–946242)	
CR1109	Δ*yceL*::FRT *kan* FRT (1123341–1124549)	
CR1110	Δ*ydeE*::FRT *kan* FRT (1619356–1620543)	
CR1111	Δ*ynfM*::FRT *kan* FRT (1667723–1668976)	
CR1112	Δ*ydhP*::FRT *kan* FRT (1734145–1735314)	
CR1113	Δ*mdtG*::FRT *kan* FRT (1113487–1114713)	
CR1114	Δ*yebQ*::FRT *kan* FRT (1908300–1909673)	
CR1115	Δ*shiA*::FRT *kan* FRT (2051667–2052983)	
CR1116	Δ*mdtD*::FRT *kan* FRT (2159488–2160903)	
CR1117	Δ*bcr*::FRT *kan* FRT (2276592–2277782)	
CR1118	Δ*yfcJ*::FRT *kan* FRT (2436964–2438142)	
CR1119	Δ*kgtP*::FRT *kan* FRT (2722470–2723768)	
CR1120	Δ*ygcS*::FRT *kan* FRT (2894555–2895892)	
CR1121	Δ*galP*::FRT *kan* FRT (3086306–3087700)	
CR1122	Δ*nanT*::FRT *kan* FRT (3369106–3370596)	
CR1123	Δ*yhhS*::FRT *kan* FRT (3608539–3609756)	
CR1124	Δ*nepI*::FRT *kan* FRT (3838572–3839762)	
CR1125	Δ*emrD*::FRT *kan* FRT (3851945–3853129)	
CR1126	Δ*mdtL*::FRT *kan* FRT (3889638–3890813)	
CR1127	Δ*proP*::FRT *kan* FRT (4328525–4330027)	
CR1128	Δ*yjhB*::FRT *kan* FRT (4502081–4503298)	
CR1129	Δ*yjiO*::FRT *kan* FRT (4565310–4566542)	
CR1130	Δ*ydhC*::FRT *kan* FRT (1737935–1739146)	
CR1131	Δ*setA*::FRT	
CR1132	Δ*setB*::FRT	
CR1133	Δ*setC*::FRT	
CR1134	Δ*ydeA*::FRT	
CR1135	Δ*mhpT*::FRT	
CR1136	Δ*yajR*::FRT	
CR1137	Δ*ybdA*::FRT	
CR1138	Δ*cmr*::FRT	
CR1139	Δ*ycaD*::FRT	
CR1140	Δ*yceL*::FRT	
CR1141	Δ*ydeE*::FRT	
CR1142	Δ*ynfM*::FRT	
CR1143	Δ*ydhP*::FRT	
CR1144	Δ*mdtG*::FRT	
CR1145	Δ*yebQ*::FRT	
CR1146	Δ*shiA*::FRT	
CR1147	Δ*mdtD*::FRT	
CR1148	Δ*bcr*::FRT	
CR1149	Δ*yfcJ*::FRT	
CR1150	Δ*kgtP*::FRT	
CR1151	Δ*ygcS*::FRT	
CR1152	Δ*galP*::FRT	
CR1153	Δ*nanT*::FRT	
CR1154	Δ*yhhS*::FRT	
CR1155	Δ*nepI*::FRT	
CR1156	Δ*emrD*::FRT	
CR1157	Δ*mdtL*::FRT	
CR1158	Δ*proP*::FRT	
CR1159	Δ*yjhB*::FRT	
CR1160	Δ*yjiO*::FRT	
CR1161	Δ*ydhC*::FRT	
CR1162	Δ*setC*::FRT *?araA*::FRT	
CR1163	Δ*cmr*::FRT *?araA*::FRT	
CR1164	Δ*ynfM*::FRT *?araA*::FRT	
CR1165	Δ*ydhP*::FRT *?araA*::FRT	
CR1166	Δ*mdtD*::FRT *?araA*::FRT	
CR1167	Δ*yfcJ*::FRT *?araA*::FRT	
CR1168	Δ*yhhS*::FRT *?araA*::FRT	
CR1169	Δ*emrD*::FRT *?araA*::FRT	
CR1170	Δ*ydhC*::FRT *?araA*::FRT	
CR1171	Δ*setC*::FRT *?araB*::FRT	
CR1172	Δ*cmr*::FRT *?araB*::FRT	
CR1173	Δ*ynfM*::FRT *?araB*::FRT	
CR1174	Δ*mdtD*::FRT *?araB*::FRT	
CR1175	Δ*yfcJ*::FRT *?araB*::FRT	
CR1176	Δ*yhhS*::FRT *?araB*::FRT	
CR1177	Δ*emrD*::FRT *?araB*::FRT	
CR1178	Δ*ydhC*::FRT *?araB*::FRT	
CR1179	Δ*setC*::FRT *?araBAD*::FRT	
CR1180	Δ*cmr*::FRT *?araBAD*::FRT	
CR1181	Δ*ynfM*::FRT *?araBAD*::FRT	
CR1182	Δ*mdtD*::FRT *?araBAD*::FRT	
CR1183	Δ*yfcJ*::FRT *?araBAD*::FRT	
CR1184	Δ*yhhS*::FRT *?araBAD*::FRT	
CR1185	Δ*emrD*::FRT *?araBAD*::FRT	
CR1186	Δ*ydhC*::FRT *?araBAD*::FRT	

a All strains are isogenic derivatives of *E. coli* K-12 strain MG1655.

b All strains are from this work unless otherwise noted.

c *E. coli* Genetic Stock Center, CGSC, Yale University.

**Table 4:** Plasmids used in this work.

Plasmids	Genotype or relevant characteristics	Source or reference^a^
pKD46	*bla* P_BAD_ *gam bet exo* pSC101 *ori(ts)*	[28]
pCP20	*bla cat cI*857 λP_R_’-*flp* pSC101 *ori(ts)*	[33]
pKD3	*bla FRT cm FRT oriR6K*	[28]
pKD4	*bla FRT kan FRT oriR6K*	[28]
pPROBE *venus*	*kan venus ori* p15a	
pTrC99A	*amp* P*_trc_ ori* (pBR322) lacl^q^	[31]
P*_araB_*-*venus*	*kan* P*_araB_*-*venus ori* p15a	
P*_xylA_*-*venus*	*kan* P*_xylA_*-*venus ori* p15a	
KK001 (pSetA)	*amp* P*_trc_ setA ori* (pBR322) lacl^q^	
KK002 (pSetB)	*amp* P*_trc_ setB ori* (pBR322) lacl^q^	
KK003 (pSetC)	*amp* P*_trc_ setC ori* (pBR322) lacl^q^	
KK004 (pYdeA)	*amp* P*_trc_ setA ori* (pBR322) lacl^q^	
KK005 (pMhpT)	*amp* P*_trc_ mhpT ori* (pBR322) lacl^q^	
KK006 (pYajR)	*amp* P*_trc_ yajR ori* (pBR322) lacl^q^	
KK007 (pYbdA)	*amp* P*_trc_ ybdA ori* (pBR322) lacl^q^	
KK008 (pCmr)	*amp* P*_trc_ cmr ori* (pBR322) lacl^q^	
KK009 (pYcaD)	*amp* P*_trc_ ycaD ori* (pBR322) lacl^q^	
KK010 (pYceL)	*amp* P*_trc_ yceL ori* (pBR322) lacl^q^	
KK011 (pYdeE)	*amp* P*_trc_ ydeE ori* (pBR322) lacl^q^	
KK012 (pYnfM)	*amp* P*_trc_ ynfM ori* (pBR322) lacl^q^	
KK013 (pYdhP)	*amp* P*_trc_ ydhP ori* (pBR322) lacl^q^	
KK014 (pMdtG)	*amp* P*_trc_ mdtG ori* (pBR322) lacl^q^	
KK015 (pYebQ)	*amp* P*_trc_ yebQ ori* (pBR322) lacl^q^	
KK016 (pShiA)	*amp* P*_trc_ shiA ori* (pBR322) lacl^q^	
KK017 (pMdtD)	*amp* P*_trc_ mdtD ori* (pBR322) lacl^q^	
KK018 (pBcr)	*amp* P*_trc_ bcr ori* (pBR322) lacl^q^	
KK019 (pYfcJ)	*amp* P*_trc_ yfcJ ori* (pBR322) lacl^q^	
KK020 (pKgtP)	*amp* P*_trc_ kgtP ori* (pBR322) lacl^q^	
KK021 (pYgcS)	*amp* P*_trc_ ygcS ori* (pBR322) lacl^q^	
KK022 (pGalP)	*amp* P*_trc_ galP ori* (pBR322) lacl^q^	
KK023 (pNanT)	*amp* P*_trc_ nanT ori* (pBR322) lacl^q^	
KK024 (pYhhS)	*amp* P*_trc_ yhhS ori* (pBR322) lacl^q^	
KK025 (pNepI)	*amp* P*_trc_ nepI ori* (pBR322) lacl^q^	
KK026 (pEmrD)	*amp* P*_trc_ emrD ori* (pBR322) lacl^q^	
KK027 (pMdtL)	*amp* P*_trc_ mdtL ori* (pBR322) lacl^q^	
KK028 (pProp)	*amp* P*_trc_ proP ori* (pBR322) lacl^q^	
KK029 (pYjhB)	*amp* P*_trc_ yjhB ori* (pBR322) lacl^q^	
KK031 (pYjiO)	*amp* P*_trc_ yjiO ori* (pBR322) lacl^q^	
KK032 (pYdhC)	*amp* P*_trc_ ydhC ori* (pBR322) lacl^q^	
KK033 (P*_setC_*-*venus*)	*kan* P*_setC_*-*venus ori* p15a	
KK034 (P*_ydeA_*-*venus*)	*kan* P*_ydeA_*-*venus ori* p15a	
KK035 (P*_mhpT_*-*venus*)	*kan* P*_mhpT_*-*venus ori* p15a	
KK036 (P*_ybdA_*-*venus*)	*kan* P*_ybdA_*-*venus ori* p15a	
KK037 (P*_ydeE_*-*venus*)	*kan* P*_ydeE_*-*venus ori* p15a	
KK038 (P*_kgtP_*-*venus*)	*kan* P*_kgtP_*-*venus ori* p15a	
KK039 (P*_cmr_*-*venus*)	*kan* P*_cmr_*-*venus ori* p15a	
KK040 (P*_ynfM_*-*venus*)	*kan* P*_ynfM_*-*venus ori* p15a	
KK041 (P*_mdtD_*-*venus*)	*kan* P*_mdtD_*-*venus ori* p15a	
KK042 (P*_yfcJ_*-*venus*)	*kan* P*_yfcJ_*-*venus ori* p15a	
KK043 (P*_yhhS_*-*venus*)	*kan* P*_yhhS_*-*venus ori* p15a	
KK044 (P*_emrD_*-*venus*)	*kan* P*_emrD_*-*venus ori* p15a	
KK045 (P*_ydhC_*-*venus*)	*kan* P*_ydhC_*-*venus ori* p15a	

a All plasmids are from this work unless otherwise noted.

The plasmids pKD3 and pKD4 were used as templates to generate scarred FRT mutants as previously described. Mutations were checked by PCR using primers that bound outside the deleted region. Prior to the removal of the antibiotic resistance marker, the constructs resulting from this procedure were moved into a clean wild-type background by P1*vir* transduction.

The plasmid pPROBE-*venus* was constructed by digesting the plasmid pQE80L-*venus* by EcoRI and NheI and cloning the fragment into pPROBE-*gfp*[tagless] digested by EcoRI and NheI. This replaced the *gfp*[tagless] by the fast-folding *yfp* variant Venus in pPROBE [30]. Venus transcriptional fusions were made by amplifying the promoter of interest and then cloning these PCR fragments into the multiple cloning site of pPROBE-*venus* . The *araBAD* promoter was amplified using primers TD088f (sequence: 5′- GGA AAG GTA CCC ATT CCC AGC GGT CG) and TD088r (sequence: 5′- GAC TAG AAT TCG CCA AAA TCG AGG CC). The *xylA* promoter was amplified using primers TD065f (sequence: 5′- GGA AAG GTA CCT CGA TCT TTT TGC CA) and TD065r (5′- GAC TAG AAT TCG CGA TCG AGC TGG TC). The PCR fragments were then digested with KpnI and EcoRI (sequences underlined) and cloned into the multiple cloning site of the pPROBE-*venus* vector. The resulting transcriptional fusions were transformed into the mutated strains containing the deletions of interest.

Plasmids overexpressing the genes of interest were constructed by cloning the respective gene into the multiple cloning site of pTrc99A under the control of a strong IPTG inducible promoter, P_trc_ [31].

### Fluorescence Assays

End-point measurements of the fluorescent reporter system were made using a Tecan Safire 2 microplate reader. 3 mL cultures were grown overnight in tryptone media at 37°C and then subcultured 1∶30 after which 0.45 mL was transferred to a single well of a polypropylene, 2.2 ml, deep, square, 96-well microtiter plate (VWR; 82006-448). Cultures were then grown at 37°C while shaking at 600 rpm on a microplate shaker (VWR). After an optical density of 0.05 was reached, the cells were induced with varying concentrations of the sugar of interest (either arabinose or xylose). Extracellular sugar concentrations ranged from 1 mM to 10 mM. In addition, strains containing the expression plasmids for the genes of interest were also induced with IPTG. Final volumes in each well were adjusted to 0.5 mL for all cultures. When the cells reached an OD of 0.4–0.5, 100 µL of the culture was transferred from the deep-well plates to black, clear-bottomed Costar 96-well microtiter plates, and the fluorescence (excitation/emission λ, 515/530 nm and OD at 600 nm (OD_600_) were measured. The fluorescence readings were normalized with the OD_600_ to account for cell density. All experiments were conducted in triplicates and average values with the standard deviations are reported.
